# A Powerful Molecular Engineering Tool Provided Efficient *Chlamydomonas* Mutants as Bio-Sensing Elements for Herbicides Detection

**DOI:** 10.1371/journal.pone.0061851

**Published:** 2013-04-17

**Authors:** Maya D. Lambreva, Maria Teresa Giardi, Irene Rambaldi, Amina Antonacci, Sandro Pastorelli, Ivo Bertalan, Ivan Husu, Udo Johanningmeier, Giuseppina Rea

**Affiliations:** 1 Institute of Crystallography, National Research Council, Monterotondo Scalo, Rome, Italy; 2 Martin-Luther-University, Plant Physiology Institute, Halle (Saale), Germany; International Atomic Energy Agency, Austria

## Abstract

This study was prompted by increasing concerns about ecological damage and human health threats derived by persistent contamination of water and soil with herbicides, and emerging of bio-sensing technology as powerful, fast and efficient tool for the identification of such hazards. This work is aimed at overcoming principal limitations negatively affecting the whole-cell-based biosensors performance due to inadequate stability and sensitivity of the bio-recognition element. The novel bio-sensing elements for the detection of herbicides were generated exploiting the power of molecular engineering in order to improve the performance of photosynthetic complexes. The new phenotypes were produced by an *in vitro* directed evolution strategy targeted at the photosystem II (PSII) D1 protein of *Chlamydomonas reinhardtii*, using exposures to radical-generating ionizing radiation as selection pressure. These tools proved successful to identify D1 mutations conferring enhanced stability, tolerance to free-radical-associated stress and competence for herbicide perception. Long-term stability tests of PSII performance revealed the mutants capability to deal with oxidative stress-related conditions. Furthermore, dose-response experiments indicated the strains having increased sensitivity or resistance to triazine and urea type herbicides with *I_50_* values ranging from 6×10^−8^ M to 2×10^−6^ M. Besides stressing the relevance of several amino acids for PSII photochemistry and herbicide sensing, the possibility to improve the specificity of whole-cell-based biosensors, via coupling herbicide-sensitive with herbicide-resistant strains, was verified.

## Introduction

Recent expansion of biosensor technology has been the direct answer to the increase in worldwide demand for cost-efficient, fast and reliable methods for monitoring chemical species, additives and xenobiotics in clinical chemistry, environmental sciences and food related processes. R&D efforts are mainly focused on the improvement of specificity, sensitivity and stability of the biosensor sensing element, which is involved in the analyte recognition process, providing easily detectable changes in its physico-chemical properties. The nature and the intrinsic properties of the bio-recognition element determine the reliability and application fields of the biodevice.

Photosynthetic microalgae are among the most preferred microorganisms for environmental monitoring and screening of food and agricultural products for hazards compounds [1], [2]. The unique features and structural constituents of the photosynthetic systems make them a suitable sensing element in the emerging biosensoristic field. This is largely due to their ability to conduct charge separation and electron transfer sensitive to the presence of different classes of pesticides and heavy metals, as well as some drugs and explosive compounds [3]–[6]. In contrast to the conventional analytical techniques, the photosynthetic biosensors offer several advantages, such as suitability for both laboratory and field applications, easy use without need of a skilled operator, minimum needs for maintenance and sample pre-treatments [7]. In addition, photosynthetic whole-cell-based biosensors allow for easy propagation and handling of the bio-recognition element, fast adaptation to changes in the working medium, possibility for regeneration and no need for extensive sample preparation [2], [5]. Nonetheless, due to laboratory manipulation, interfacing with transducer elements, or repeated cycles of operational activity, the photosynthetic microorganisms can undergo a reduction of the specific performance and/or half-life. As a consequence, the storage- and operational-stability of the bio-sensing element is a crucial point that should be addressed. Different approaches have been adopted to increase the stability of the biological elements and preserve their functionality. In the case of whole-cell-based biosensors, entrapment and immobilisation of the microorganisms in inert organic matrices are among the most widely used methods [2], [8].

Although biosensor technology is a winning alternative to the classical quantitative methods for fast and field-based pre-screenings, these biodevices are still challenged by the identification of a single analyte into the complex matrix of a real sample. The research for biosensor specificity improvement concerning microalgae is focused on screening for sensitive microalgae species [9], [10], selection of microalgal resistant genotypes [5], or generation of genetically modified species [7], [11]. In this respect, the use of biodevices exploiting an array of several bio-recognition elements specifically sensitive or resistant to a certain toxic compound represents a promising strategy.

Many biosensors, using photosynthetic recognition elements, specifically exploit the inhibition of PSII photochemical reaction in presence of different toxins. The PSII is a multiple subunit protein-chlorophyll complex responsible for the light-driven water oxidation; its core is assembled by the D1 and D2 proteins, which host all cofactors involved in the photochemical electron transport reaction [12]. A typical example of contaminants detected by PSII-based biosensor devices are the photosynthetic herbicides, which are widely used in the agricultural practice. Due to their extensive exploitation, high persistence and mobility, some of these compounds are widespread water and food contaminants, bringing about considerable ecological damage, as well as posing a threat for human health [6]. These toxic substances can be straightforwardly detected by PSII, since they interact with the D1 core complex protein coupling to the binding site of the secondary electron acceptor, the plastoquinone (Q_B_) [13]. The herbicides successfully compete with the native electron acceptor due to their high binding affinity, blocking the PSII electron transport rate in a concentration dependent manner. The presence of PSII herbicides is easily detectable by the rapid decline of the primary plastoquinone (Q_A_) reoxidation rate, reflected by changes in the chlorophyll *a* fluorescence, reduction of oxygen production and the photosynthetic electron transport rate [7], [8], [14].

In the present study, we have used the unicellular green alga *Chlamydomonas reinhardtii*, which has become one of the most-commonly exploited model microorganisms in various fundamental and applied studies. It offers the benefits of high growth rate and easy cultivation of the microorganisms, and the ability to perform post-transcriptional and post-translational modifications distinctive for the higher plants. All these advantages and the complete annotation of its genome [15] turned *C. reinhardtii* into a robust platform for synthesis of bioactive compounds, recombinant protein expression [16], [17], biomass and biofuels production [18], [19], and a main protagonist in bioremediation and biological sensing research fields [8], [11], [20], [21]. In particular, the rational design and *ad hoc* production of *C. reinhardtii* mutants for biosensor purposes has been truly facilitated by the advances in its chloroplast transformation and resolution of PSII crystal structure [7], [11], [20], [22].

This research was focused on the identification and characterisation of novel bio-recognition elements suitable for herbicide sensing by whole-cell-based biosensors. In particular, it aimed at overcoming core limitations affecting biosensor performance due to inadequate stability and sensitivity of the biological component. Coupling random mutagenesis targeted to the PSII D1 protein with exposure to ionizing radiation as selection pressure, we aimed to isolate several radical-stress tolerant mutants, which also had modified sensitivity to different classes of herbicides. The final objective was to unravel the possibility to develop biosensor arrays constituted by herbicide-sensitive and herbicide-resistant strains. The results discussed in this study demonstrated the suitability of the adopted directed evolution approach for identification of mutants with enhanced stability under free radocal-assosiated stress conditions, and their use for improving the selectivity of biosensor devices.

## Results and Discussion

The *in vitro* directed evolution strategy is a unique molecular tool used to generate genetic diversity in a specific *locus*, mimicking and speeding-up the natural evolution process. This approach is particularly useful to evidence crucial sequences in genes characterised by low genetic variability, and when coupled to a functional selection allows for the easy discovery of novel traits. Exploiting directed evolution, we produced a random mutant library of *C. reinhardtii* with improved stability to oxidative stress-related conditions. The approach was targeted to the highly evolutionary conserved D1 PSII core protein, hosting a binding niche for the secondary electron acceptor Q_B_ and most of its photosynthetic herbicides competitive inhibitors. The newly isolated D1 random mutants were characterised under physiological and stressful conditions and their relevance for biosensor purposes was demonstrated by testing the strain's resistance/sensitivity toward triazines and urea type herbicides.

### Generation and selection of radical tolerant D1 random mutants of *C. reinhardtii*

Random mutagenesis was performed by ep-PCR into a 400 bps nucleotide sequence of the *psb*A gene. This sequence encodes a D1 functionally important region (Ala153-Ala294) including the tyrosine residue at position 161, the primary electron donor for P_680_ and the transmembrane helices IV and V building up the Q_B_ binding niche [12]. Following biolistic chloroplast transformation, positive D1-transformants were selected for their competence for photoautotrophic growth. This method brought the advantage of obtaining marker-free transgenic strains, avoiding introduction of any foreign or antibiotic-resistant genes in the genome. Further on, the library of photosynthetically competent D1 mutants were utilized to isolate strains with improved tolerance to free-radical-associated stress conditions by exposures to additional selective pressure, such as neutron and proton bombardments. One of the main impact that these high-energy particles could cause to any biological material is derived from the secondary generated radical species and the subsequent oxidative chain reactions [23]. Table 1 lists mutants, which survived to the ionizing radiation exposures and were further analysed in the present study. The power of this approach to isolate strains with an improved tolerance to radical associated stress conditions has been demonstrated in a previously published work [24]. The common trend observed in the D1 aminoacidic substitutions was the replacement of less polar by more polar amino acids. In other words, the applied selection pressure forced replacement of residues, more sensitive to oxidative damage, with less sensitive ones. The relevance of this finding in the cellular context was demonstrated by *de novo* produced D1 site-directed mutants of *C. reinhardtii* carrying some of the aminoacidic substitutions selected by the ionizing radiation experiments. In comparison with the referent IL, the new strains displayed reduced photosynthetic performance under growth conditions and increased electron transport efficiency and oxygen evolution capacity in stressful high-light conditions. These findings enriched the abundant evidence present in the literature for the central role of the aminoacidic composition and transcriptional and post-translational regulation of the D1 protein in ruling the whole photosynthetic process under variety of environmental conditions [25].

**Table 1:** Amino acid substitutions and their localization in the D1 protein of the analysed *C. reinhardtii* random mutants survived after neutrons and protons exposures.

Strain	Amino acid substitution		Localization of the
	wild type→mutated		mutation in D1 protein
L159M	leucine	methionine	near to Tyr_161_
L159I/I184V	leucine	isoleucine	near to Tyr_161_
	isoleucine	valine	near to EOC
P162S/F211S	proline	serine	near to Tyr_161_
	phenylalanine	serine	helix IV of D1
I163T/I224F	isoleucine	threonine	near to Tyr_161_
	isoleucine	phenylalanine	helix IV of D1
M172L	methionine	leucine	near to EOC
M172T	methionine	threonine	near to EOC
S177P	serine	proline	near to EOC
F197C/F285C	phenylalanine	leucine	helix IV of D1
	phenylalanine	leucine	helix V of D1
L200I	leucine	isoleucine	helix IV of D1
G207S	glycine	serine	helix IV of D1
F274Y	phenylalanine	tyrosine	helix V of D1
I281T	isoleucine	threonine	helix V of D1

**Figure 1:**
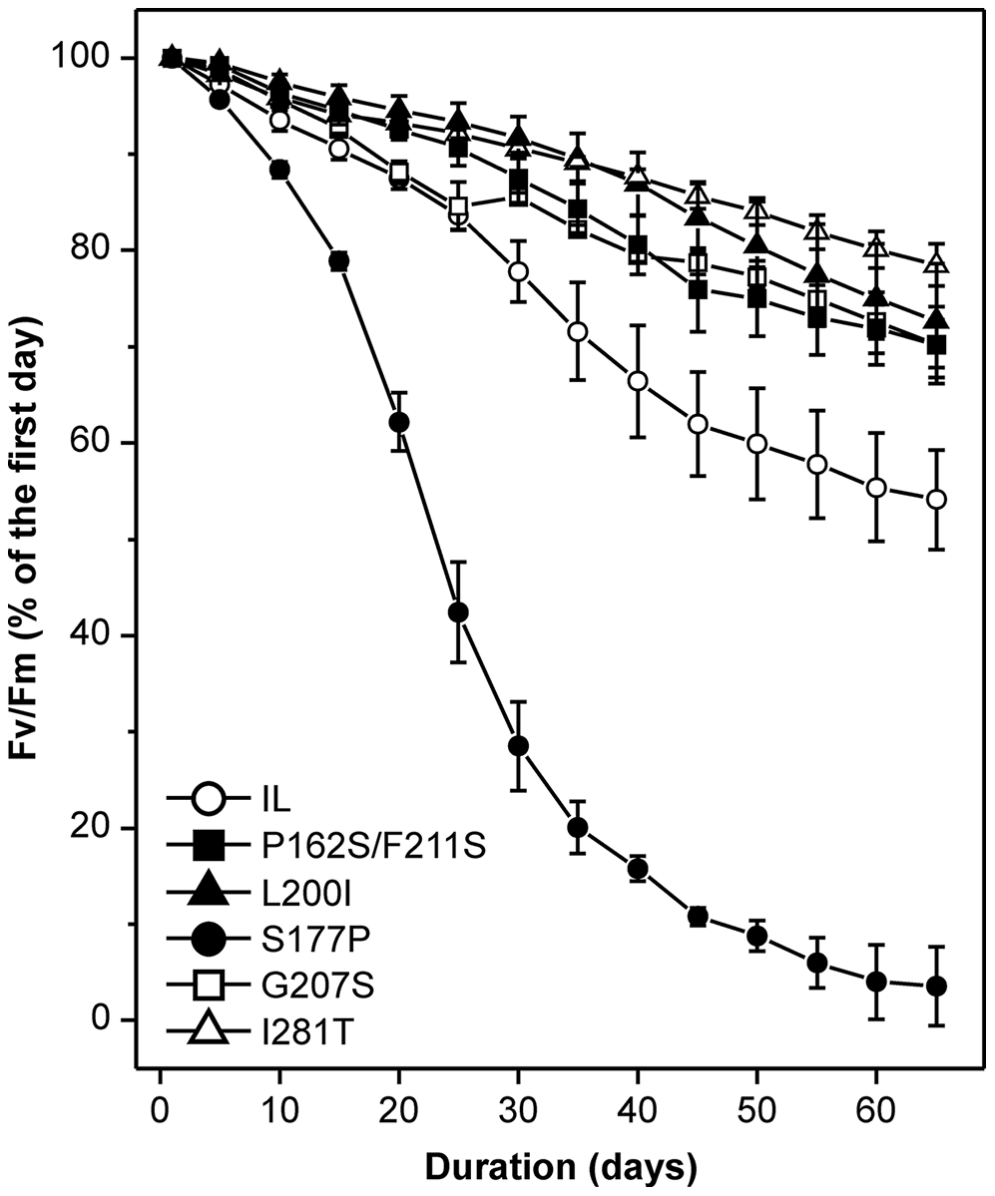
Long-term stability test of *C. reinhardtii* strains under oxidative stress conditions. The resistance of the strains is expressed as a reduction of their maximum quantum yield of the photochemical reaction (F_v_/F_m_) over time. Fluorescence measurements were performed hourly during the 7 h light/17 h dark photoperiod; herein are reported F_v_/F_m_ values measured at the end of the light phase. Average values from two independent experiments are presented, ±SE, n = 4.

In the present work the tolerance of the D1 random mutants of *Chlamydomonas* toward radical-associated conditions was also questioned by the following long-term stability test. Hence, the algal cultures were exposed to low temperature (13°C) and low light intensity (20 µmol m^−2^ s^−1^) and their PSII performance was examined hourly for approximately two months (Figure 1). The low temperature stress slows down all catabolic reactions, decreasing the demand of absorbed energy and impeding the photosynthetic electron transport. These phenomena lead to accumulation of long-lasting reduced forms of the PSII primary quinone (Q_A_) [26]. As a result, the probability for oxygen radical species generation and oxidative damage increases considerably [27]. The optimal growth temperature for most *Chlamydomonas* species varies in the range of 20–25°C and few of them could tolerate temperatures as low as 15°C [28]. Moreover, it has been shown that short (hours) or long (days) treatment with low temperature stress could reduce the overall fitness and viability of the *Chlamydomonas* cells, the chlorophyll stability and membrane integrity [29], [30]. After 65 days exposure, the D1 mutants P162S/F211S, L200I, G207S and I281T preserved more that 70% of their initial photosynthetic activity, as determined by the F_v_/F_m_ ratio, representing the maximum quantum yield of PSII photochemical reaction (Figure 1). In contrast, under these non-lethal stress conditions, the reference strain IL maintained only 54±5% of its initial PSII performance. Similarly to IL, the M172L, F197L/F285L and L159M mutants retained 66±2%, 57±3% and 55±3% of their initial F_v_/F_m_ values, respectively (Table S1). On the contrary, the mutants F274Y, S177P and M172T were very sensitive to the applied stress conditions and at the end of the treatment they were able to keep only 25±1%, 4±2% and 0% of their F_v_/F_m_, respectively (Figure 1 and Table S1). Indeed, the mutant M172T did not resist the experimental conditions more than 30 days. I281T stood out against all strains as the most resistant to radical-related stress conditions, preserving 76±2% of its F_v_/F_m_ value even after 110 days treatment (IL retained only 46±4%) (Table S1). When maintained at physiological temperature the average reduction of PSII performance in the same strains did not exceed 20±5%.

In addition, the tolerance to radical-associated stress conditions of some of these strains was verified by free radical scavenging capacity assay, based on the cell extract potential to reduce the stable DPPH radicals present in a reaction mixture. The cell extracts of the tolerant strains P162S/F211S, L200I, G207S and I281T showed higher antioxidant power in comparison with the reference IL. This trait was estimated by the extract concentration (mg dry weight mL^−1^) necessary to reduce 50% of the DPPH free radicals (Table S2). Moreover, the radical scavenging capacity assay on the strain F274Y, characterized by low performance in the long-term stability test (Table S1), showed lower ability to reduce the DPPH free radicals compared to IL. These results clearly demonstrate the improved ability of the selected tolerant strains to deal with radical associated stress conditions. Hereafter in this study, in-depth photosynthesis analyses were performed only on mutants showing high photosynthetic stability in stressful conditions (Table 2); in particular, mutants demonstrating reduction of F_v_/F_m_ value lesser than 50% over a 2 months period, were characterised in physiological conditions and in the presence of herbicides.

**Table 2:** Physiological parameters of the reference strain IL and D1 random mutants of *C. reinhardtii* selected for their PSII long-term stability under oxidative stress-related conditions.

Strains	Total Chl	F_v_/F_m_	*1-V_J_*	O_2_ evolution
	µg mL^−1^			µmol O_2_ mg Chl^−1^ h^−1^
IL	7.7±0.9	0.773±0.002	0.548±0.003	92±3
L159I/I184V	3.5±0.6	0.691±0.011	0.499±0.008	112±4
P162S/F211S	3.7±0.0	0.664±0.022	0.518±0.017	120±3
M172L	2.5±0.0	0.731±0.006	0.511±0.012	112±3
F197L/F285L	3.3±0.8	0.665±0.011	0.472±0.009	141±3
L200I	3.9±0.4	0.686±0.009	0.537±0.006*	110±6
G207S	3.7±0.2	0.731±0.003	0.556±0.009*	129±4
I281T	3.9±0.3	0.707±0.005	0.545±0.004*	115±3

The total chlorophyll content, the maximum quantum yield of PSII photochemical reaction (F_v_/F_m_), the efficiency of the electron transport through PSII primary and secondary quinones (*1-V_J_*) and the oxygen evolution rate (measured at 350 µmol m^−2^ s^−1^ light intensity) were determined in cell cultures in exponential growth phase (*OD_750_* = 0.45±0.02). Average values are presented, ±SE, 4<*n*<12; the asterisks indicate values that are not significantly different from the IL strain at p≤0.05 (Mann-Whitney U Test).

### Physiological characterization of selected mutants

**Figure 2:**
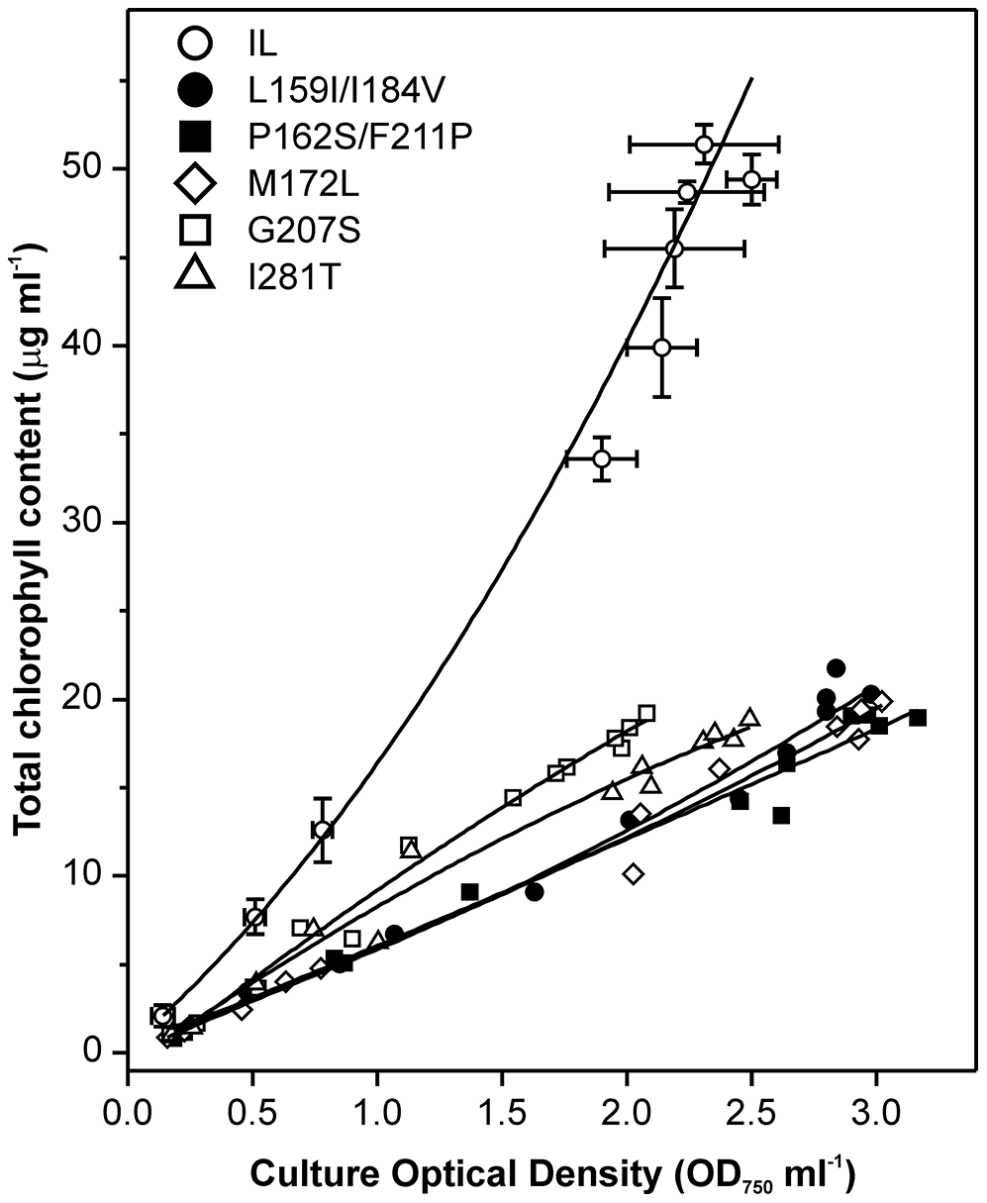
Comparison of the *C. reinhardtii* strains culture chlorophyll and optical density during growth. The development of the reference strain, IL, and D1 random mutants is presented as a ratio of the total chlorophyll mL^−1^ and the corresponding OD_750_ mL^−1^. The cell cultures were grown for a period of 88 h in TAP medium under continues illumination of 50 µmol m^−2^ s^−1^ at 24°C and 150 rpm agitation. Average values from four experiments are presented, ±SE, n = 4. For the sake of clarity the standard error bars of the mutants values are omitted.

The growth of selected *Chlamydomonas* strains was characterized measuring culture optical density and chlorophyll content for a period of 88 h (Figure S1). During all growth phases, the *OD_750_* was similar among the mutants and a little bit higher in comparison with the reference strain IL during the late growth phase. On the contrary, the D1 mutants accumulated much lower amount of chlorophyll per mL than IL. In the exponential growth phase, the mutant total chlorophyll content was half compared to IL (Table 2). This deviation gradually increased with the time resulting in very low chlorophyll/*OD_750_* ratios for the mutants in the late growth phase (Figure 2).

Amino acid replacements in the D1 selected region influenced the PSII function, lowering the mutants maximum photochemical quantum yield as reported in Table 2. On the other hand, the efficiency of the mutants electron transport between the primary (Q_A_) and secondary (Q_B_) quinones in PSII (*1-V_J_*) was lower, but still similar to IL. This finding suggested that even though the electron transfer through the PSII was altered by the amino acid substitutions, no severe interruption of the PSII functioning occurred in the selected D1 mutants. In support of this statement is the higher capacity of the mutants to evolve oxygen under saturating light intensity (350 µmol m^−2^ s^−1^) in comparison with the reference strain (Table 2). Previous experiments showed that a light intensity of 200 µmol m^−2^ s^−1^ saturates the photosynthetic reactions in IL and a further increase in radiation intensity was not correlated to an increased in the amount of the oxygen released [24]. In contrast to IL, under saturating light the D1 mutants were able to produce from 20% (L200I) up to 54% (F197L/F285L) more oxygen per mg chlorophyll than the reference strain.

### Relevance of selected mutants as bio-recognition elements

**Figure 3:**
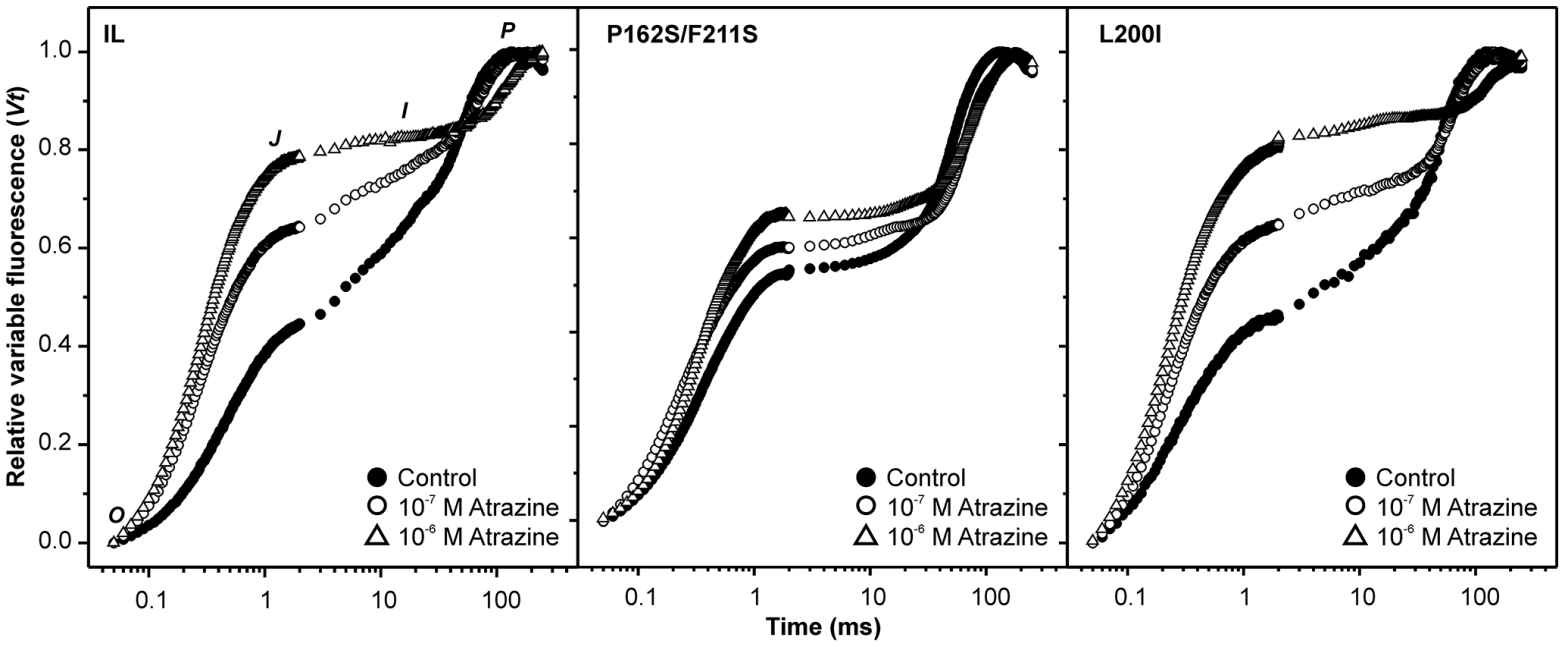
Polyphasic chlorophyll fluorescence transient under different atrazine concentrations. The fluorescence transients of reference strain IL and P162S/F211S and L200I mutants are presented as curves of the relative variable fluorescence, *V_t_* = (F_t_-F_0_)/(F_m_-F_0_). Typical *V_t_* curves for each strain and herbicide treatment are presented; the characteristic points *O-J-I-P* are also indicated.

The response of the selected strains to triazines and urea type herbicides was studied by chlorophyll fluorescence induction curves (*O-J-I-P*) [31], normalised by the maximum variable fluorescence (*F_v_ = F_m_-F_0_*), as reported in Materials and Methods. This normalisation allows for a direct comparison between the samples, excluding any absorbance or heat dissipation differences (Figure 3). The relative variable fluorescence at the characteristic point *J* (*V_J_*) gives a measure of relative amount of the reduced Q_A_. Therefore, *1-V_J_* represents the fraction of oxidised Q_A_ or the efficiency by which the electron is transferred from Q_A_ to Q_B_ [32]. Using this approach, we evaluated the sensitivity of the newly generated strains to two different classes of PSII herbicides. In the absence of inhibitor, the *V_J_* registered in the mutant P162S/F211S is higher than in the IL strain, thus reflecting the lower efficiency of the electron transport through PSII (*1-V_J_*) under physiological conditions. In the presence of PSII herbicides, the Q_B_ binding site is permanently occupied by the inhibitor and electron transport does not extend beyond Q_A_. The accumulation of reduced forms of Q_A_ is proportional to the herbicide concentration and can be directly evaluated by the increase of the variable fluorescence level at point *J* (Figure 3), making the parameter *1-V_J_* one of the most sensitive for herbicide toxicity detection [10], [14], [20].

**Figure 4:**
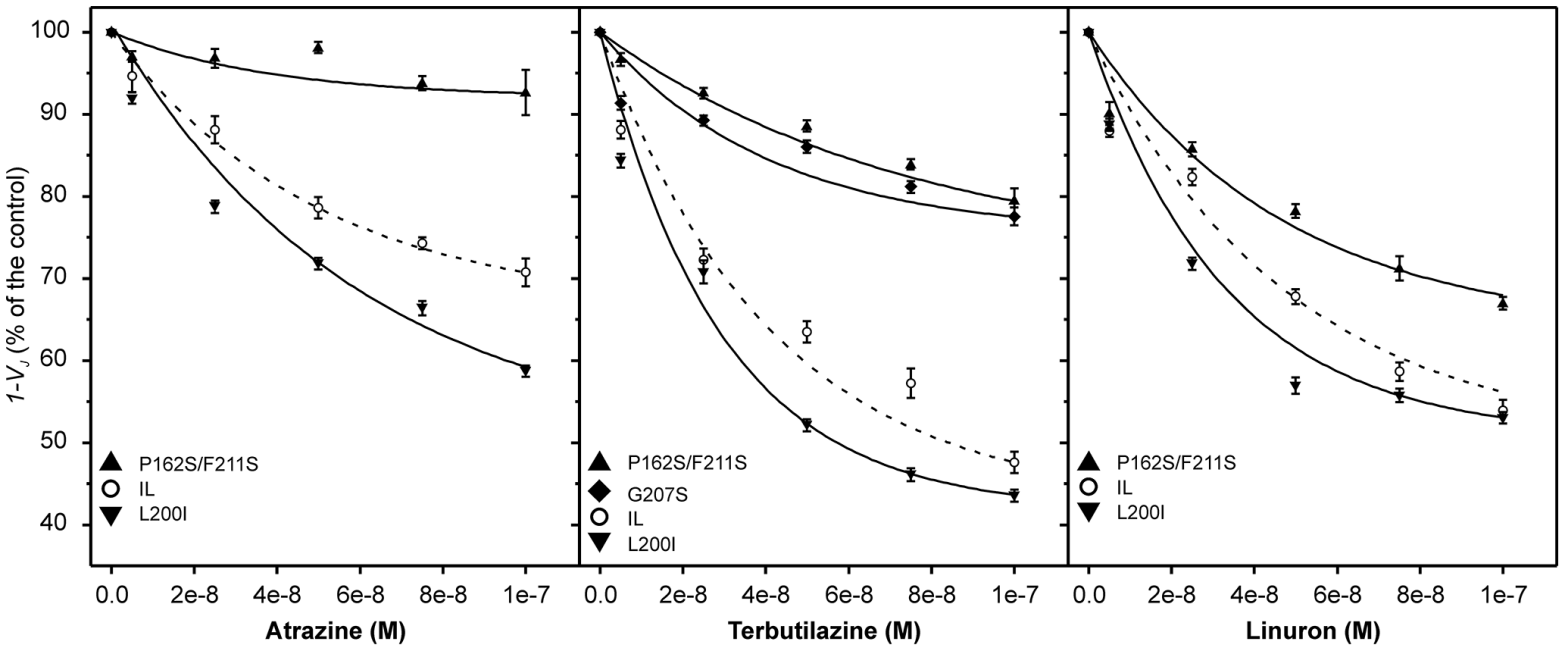
Herbicide dose-response curves of PSII electron transport efficiency. Fluorescence transients of the reference strain IL and D1 random mutants of *C. reinhardtii* were registered after 10 min of incubation with increasing concentrations of atrazine, terbutilazine or linuron. The parameter *1-V_J_* was calculated as *1-V_J_ = *1-(F_2ms_-F_0_)/(F_m_-F_0_) according to Strasser et al. [32]. Average values from three independent experiments are presented, ±SE, n = 6–9.

The herbicide sensitivity or resistance of the newly characterized strains, was assessed by measuring the herbicide-induced reduction of the parameter *1-V_J_* (Figure 4), and evaluated by the *I_50_* and corresponding R/S values (Table 3). R/S>1.0 points out herbicide resistance, while R/S<1.0 refers to herbicide “supersensitivity” [33]. In this study, only 0.9≤R/S≥1.1 values were considered to indicate significant changes in the mutants tolerance to photosynthetic herbicide. The mutants herbicide sensitivity was evaluated also by the LOD parameter, which represented the minimal analyte concentration inducing significant reduction of the algal PSII electron transport efficiency (Table 4). The L200I mutation conferred supersensitivity to both triazine and urea type herbicides. In this mutant, the R/S values were 0.7 for atrazine, 0.6 for terbuthylazine and 0.8 for linuron. The L200I LOD values for terbuthylazine and linuron were 3-fold and 4-fold lower than IL, respectively, confirming the supersensitivity of this mutant. The substitution of methionine 172 with leucine did not cause significant changes in *I_50_* and R/S values for the tested herbicides, but led to approximately 3 times lower LOD values for atrazine and linuron compared to the reference strain. This result could be ascribed to the M172L supersensitivity to very low herbicide concentrations (within nano-molar range), which might be advantageous for its use as biosensor bio-recognition element. In contrast, the double mutant P162S/F211S showed reduced affinity to all tested inhibitors; its herbicide resistance decreased in the order atrazine (R/S = 8.2)> terbuthylazine (R/S = 6.2)> linuron (R/S = 1.9). As evident from the LOD values, the P162S/F211S minimal detectable concentration for the tested herbicides declined in the same order (Table 4). While the D1 aminoacidic substitutions in L200I and P162S/F211S strains resulted in an increase in their sensitivity or resistance to the tested inhibitors, the replacement of isoleucine 281 with threonine (I281T strain) determined opposed responses to triazines and ureas. The I281T mutant was supersensitive to atrazine and terbuthylazine (R/S = 0.8) and resistant to linuron (R/S = 1.2), even though both triazines and urea type herbicides target the same D1 amino acid residue (S264) in the Q_B_ binding niche [13]. In contrast, the G207S mutant, hosting a serine instead of a glycine, showed reduced affinity to terbuthylazine and linuron (R/S values 4.4 and 1.2 respectively), while its *I_50_* for atrazine was very similar to the inhibition constant of the reference strain (R/S = 0.9). It has been previously demonstrated that the same point mutations in D1 aminoacidic composition, which cause resistance towards one class of herbicides, can cause a supersensitivity to other classes of photosynthetic inhibitors [22], [33]. This finding was explained by the possibility for different interactions between the herbicide molecules and the amino acids lining the cavity of the Q_B_ binding pocket, although the inhibitors interface common regions of the binding pocket.

**Table 3:** Herbicides toxicity effects on the PSII electron transport efficiency (*1-V_J_*) of IL and selected D1 random mutants, and corresponding R/S values.

Strains	Atrazine		Terbuthylazine		Linuron	
	*I_50_*×10^−7^ M	*R/S*	*I_50_*×10^−7^ M	*R/S*	*I_50_*×10^−7^ M	*R/S*
	(±SE)		(±SE)		(±SE)	
IL	2.4±0.11	/	1.0±0.08	/	1.1±0.05	/
P162S/F211S	19.7±1.57	8.2	5.9±0.26	6.2	2.0±0.17	1.9
M172L	2.2±0.03*	0.9	1.1±0.02*	1.1	1.1±0.06*	1.0
L200I	1.8±0.06	0.7	0.6±0.04	0.6	0.9±0.06	0.8
G207S	2.1±0.09*	0.9	4.3±0.18	4.4	1.3±0.09	1.2
I281T	2.0±0.07	0.8	0.7±0.02	0.8	1.3±0.05	1.2

The *I_50_* is the molar herbicide concentration, which induces 50% inhibition of the parameter *1-V_J_*. The R/S were calculated as ratios of mutant *I_50_* and *I_50_* of the reference strain; the asterisks indicate values that do not differ significantly from the IL strain at p≤0.05 (Mann-Whitney U Test). We considered that R/S<0.9 values define increased herbicide sensitivity, R/S>1.1 – increased herbicide resistance and 0.9≤R/S≤1.1 – no significant alterations in the mutants herbicide sensitivity relative to the reference strain.

**Table 4:** Limits of detection of IL and selected D1 random mutants to three different herbicides, representing the lower herbicide molar concentration inducing detectable inhibition of strains PSII electron transport efficiency.

Strains	Limit of detection (×10^−9^ M)		
	Atrazine	Terbuthylazine	Linuron
IL	10	4	6
P162S/F211S	75	24	8
M172L	3	3	3
L200I	8	1	2
G207S	9	23	5
I281T	5	8	8

Extensive literature proved the relevance of aminoacidic substitutions in the Q_B_ binding niche of D1 protein in the modulation of the herbicide response in higher plants, algae and cyanobacteria. Most of these mutations were localised in the D1 primary structure encompassing amino acids from phenylalanine 211 to leucine 275 [12], [22], [33], [34]. In *C. reinhardtii*, it has been demonstrated that aminoacidic mutations outside this region can also modify the herbicide sensitivity, leading to hypothesise long-range effects in the arrangement of the D1 Q_B_ hosting cavity [22]. In the present study, excluding the Phe 211, all the selected mutations were localised outside the Q_B_ binding niche (M172L, L200I, G207S and I281T), and were not previously described. Comparative analyses of triazine and urea type herbicides binding indicated *I_50_*values in the same order of magnitude of those previously reported by Wilski et al. for similar D1 mutations in *C. reinhardtii* [22].

Molecular dynamics simulations evidenced an active role of Phe 211 in the atrazine binding to the Q_B_ pocket [6], while an atomic structure resolution of cyanobacterial PSII provided evidence on terbutryn binding to the same residue [13]. In *C. reinhardtii*, aminoacidic replacements of Phe 211 with alanine, glycine, threonine or isoleucine were usually associated to additional mutations, and were responsible for an increased resistance to different classes of herbicides. The literature reported R/S values were similar or somewhat lower compared to those determined in our study for the P162S/F211S strain [33]. Similar herbicide responses and R/S values were also reported in *Synechococcus* PCC7002 and *Synechocystis* PCC6714 AzI and AzV mutants [33].

The assembly of herbicide resistant and sensitive genotypes on multiarray transducers or biosensor platforms could be a very useful strategy to increase the specificity of these analytical devices. For instance, when P162S/F211S, G207S and L200I strains are exposed to terbuthylazine, only L200I will demonstrate fast and high reduction in its photosynthetic activity, while in the presence of atrazine only the double mutant P162S/F211S will maintain high PSII electron transport efficiency. In a recent review, Hernández-Allica et al. [5] described the successful development of a fibre optic biosensor based on the comparative response of herbicides sensitive and resistant genotypes. This data reiterates the benefits of using resistant strains to improve the specificity of whole-cell based biosensors. In addition, we proposed a molecular bio-engineering strategy for fast and efficient generation of resistant genotypes.

Using a conventional instrument for prompt fluorescence registration, very short incubation time and selected *Chlamydomonas* mutants, it was possible to distinguish nano-molar concentrations of triazines and urea type herbicides that are very close to the limits imposed by the European legislation [6]. A significant increase of the proposed biomediators sensitivity can be expected when the newly generated bio-recognition elements are combined with last generation biosensor devices [35], or microfluidic chip with organic light emitting diode and photodetector [8]. In addition, the biosensors exploiting multiarray platforms [1], [5], [7], [9] offer the possibility to test the same sample by several sensing elements working in series. Coupled with *ad hoc* designed or selected sensitive and resistant strains this approach can significantly contribute to increasing whole-cell-based biosensor's specificity.

### Conclusions

Random mutagenesis targeted at the PSII D1 protein combined with an *in vitro* directed evolution strategy succeeded in the selection of *Chlamydomonas* strains with reduced susceptibility to free-radical related stressful conditions and altered herbicide sensitivity. Among the twelve D1 random mutants which survived to protons/neutrons exposures, seven demonstrated high ability to maintain functional photosynthetic apparatus for more than 65 days. Out of the tolerance strains, five showed an increased sensitivity/resistance to herbicides, and revealed to be promising bio-recognition elements. Furthermore, the significance of new D1 aminoacidic residues for the PSII electron transfer and herbicide sensing was demonstrated and the perspectives for improving whole-cells based biosensors specificity and sensitivity have been discussed.

## Materials and Methods

### Generation and selection of the *Chlamydomonas* strains

The libraries of PSII D1 protein mutants of *C. reinhardtii* were produced by random mutagenesis of the *psb*A gene, coupled with biolistic transformation of the Del1 deletion mutant as previously described [24]. Briefly, a pool of randomly mutated *psb*A gene-fragments was produced by error-prone (ep) PCR using a pSH5 plasmid as a template, which hosts an intronless *psb*A gene [36]. Positive transformants were selected for their photoautotrophic growth on high salt (HS) medium. In order to isolate algal strains tolerant to free-radical-induced oxidative stress, a coctail of photoauthotrophycally competent D1 random mutants were subjected to an additional selection pressure applied by bombardments with protons and neutrons [24]. The IL strain of *C. reinhardtii* with intronless *psb*A gene [37] was utilized as a reference strain in all the experiments evaluating the functionality and herbicide sensitivity of the newly produced D1 random mutants.

### Growth conditions and media

The *C. reinhardtii* strains were grown in Tris-acetate-phosphate (TAP) or HS media [28] under continuous illumination of 50 µmol m^−2^ s^−1^, 24±1°C and agitation (150 rpm). When necessary, the media were solidified with 1.5% agar. In all analyses algae cultures in exponential growth phase (optical density, *OD_750_* = 0.45±0.02) were used.

### Physiological characterisation of the selected bio-recognition elements

The growth rate of the strains was tracked by measuring the culture *OD_750_* and total chlorophyll content during an 88 hour period as described previously [24].

PSII performance was evaluated by registering the chlorophyll *a* fluorescence induction curves at 24°C in TAP liquid cell cultures by the Plant Efficiency Analyser (PEA, Hansatech Instr. Ltd, Norfolk, UK). The fluorescence transient was induced by 3 s saturated pulse (600 W m^−2^) with peak at 650 nm. Before each measurement the samples were kept for 10 min in dark in order to register PSII performance in dark adapted steady state. To allow for direct comparison between the different samples the OJIP fluorescence transients were normalised to the maximum variable fluorescence (F*_v_ = *F_m_-F_0_), providing the curves of the relative variable fluorescence *V_t_ = *(F_t_-F_0_)/(F_m_-F_0_). The maximum quantum yield of PSII photochemistry was calculated as F_v_/F_m_ = (F_m_-F_0_)/F_m_; the efficiency of the electron transport between PSII primary (Q_A_) and secondary (Q_B_) quinone electron acceptors was evaluated by the parameter *1-V_J_*, where *1-V_J_* = 1-(F_2ms_-F_0_)/(F_m_-F_0_) [32]. F_0_, F_m_ and F_2ms_ are the fluorescence level at 50 µs, the maximum fluorescence and the fluorescence level at 2 ms after the onset of the illumination, respectively. The measurements were performed on at least three separate cultures, with an average of four repetitions for each culture.

The oxygen evolution capacity was measured at 24°C by Clark-type oxygen electrode (Chlorolab 2, Hansatech, Instr. Ltd, Norfolk, UK). The oxygen evolution rate was determined under saturating illumination (350 µmol m^−2^ s^−1^), continuous stirring and in presence of 10 mM NaHCO_3_, as additional carbon source [38]. To compare the photosynthetic capacity between the different samples, the same amount of chlorophyll (15 µg mL^−1^) was loaded into oxygen electrode chamber. Each strain was assayed in three separate cultures, with three repetitions for each culture.

### Long-term stability test

The strains long-term resistance test to stressful conditions was performed using the Photo II fluorometer [39]. For each strain calculated amount of cell culture was harvested by weak centrifugation to reach a final *OD_750_* = 9 and layered on TAP agar medium solidified in the instrument containers. The algal cells were exposed to 20 µmol m^−2^ s^−1^ light intensity with 7 h light/17 h dark photoperiod at 13±1°C. Photosynthetic performance of the cultures was followed for approximately 2 months by hourly registration of chlorophyll *a* fluorescence induction curves. Each fluorescence measurement was performed on 15 min dark-adapted sample by a 6 s pulse of red light.

### Herbicides treatment of *Chlamydomonas* cell cultures

The triazine herbicides atrazine (2-chloro-4-(ethylamine)-6-(isopropylamine)-s-triazine) and therbuthylazine (6-chloro-*N*-(1,1-dimethylethyl)-*N*′-ethyl-1,3,5-triazine-2,4-diamine) and the phenylurea herbicide linuron (1-methoxy-1-methyl-3-(3,4-dichlorophenyl)urea) were purchased from Sigma Aldrich (USA). 50 mM and 5 mM herbicide stock solutions were prepared in 100% methanol and water-diluted to prepare the working solutions. The experimental data set for each tested herbicide included control and at least nine different concentrations (10^−9^ M, 5×10^−9^ M, 2.5×10^−8^ M, 5×10^−8^ M, 7.5×10^−8^ M, 10^−7^ M, 2.5×10^−7^ M, 5×10^−7^ M, 10^−6^ M). In order to obtain at least 90% PSII electron transport inhibition in some of the mutants the herbicide dose-response curves were registered up to 2×10^−5^ M herbicide concentration. For the treatment, algal cultures (15 µg chlorophyll mL^−1^) were incubated with herbicide for 10 min under 50 µmol m^−2^ s^−1^ irradiation, 24°C and constant stirring. The herbicides toxicity was evaluated following the inhibition level of the parameter *1-V_J_*, measured and calculated as described above. The maximum final concentration of the methanol in algal samples was 0.02% and by itself did not alter the algal performance (data not shown). The effect of each herbicide was tested by performing the dose-response curve on three independent biological cultures, with at least two repetitions for each culture.

### Evaluation of the bio-recognition elements herbicide response

The inhibition constant *I_50_* (herbicide concentration required for 50% inhibition of the parameter *1-V_J_)* and the limit of detection (LOD) were determined for all herbicides and examined *Chlamydomonas* strains. The inhibition of PSII electron transport efficiency at increasing herbicide concentration was calculated as *100-(1-V_J_)_herbicide_/(1-V_J_)_control_*. The data points followed a typical Langmuir isothermal ligand binding curve that can be fitted by the equation (1): *y = *(*I_MAX_×x)/(I_50_+x)*, where *x* and *y* stand for the molar herbicide concentration and the percentage of inhibition of the parameter *1-V_J_* respectively; *I_50_* is the herbicide concentration required for 50% inhibition of *1-V_J_* and *I_MAX_* is the maximum registered inhibition of *1-V_J_*. *I_50_* and *I_MAX_* are free parameters attained by the fitting of the experimental data. Since the experimentally obtained reduction of the parameter *1-V_J_* did not exceed 90%, the *I_50_* achieved from the fit of the dose-response curve referred to an *I_MAX_* value significantly lower than 100% inhibition. Therefore, the actual value of *I_50_* was calculated by solving the equation (1) for y = 50% using the *I_50_* and *I_MAX_* obtained from the fit. The herbicide sensitivity or resistance was assessed also by R/S values calculated as a ratio of mutant *I_50_* and *I_50_* of IL strain. As a statistical parameter, the LOD represents an estimation of the lowest concentration of herbicide analyte that may be experimentally detected with a certain confidence level. It was calculated using the actual value of *I_50_* according the equations reported by Maly et al. [40]: LOD = (2.6×σ×*I50*)/(100−2.6×σ), where σ is the mean standard error of the measurements, while 2.6×σ indicates the confidence interval in which there is a 99% probability of finding measurement results following the assumption of a normal distribution.

### Statistical analyses

Presented data are means of at least three independent experiments with at least two repetitions each. The differences between the reference strain and each of the D1 mutants were assessed by a non-parametric Mann-Whitney U test for comparison of two independent samples. The statistical significance of the differences was evaluated at p≤0.05 values.

## Supporting Information

Figure S1 **Growth rate curves of the reference strain IL and D1 random mutants of** ***C. reinhardtii*** **selected for their PSII long-term stability under oxidative stress-related conditions.** The time courses of cell culture growth were followed for a period of 88 h under growth conditions by measuring: **A**) culture optical density (OD_750_) and **B**) total chlorophyll content (µg/ml). Average values from four different cultures are presented, ±SE, n = 4.
(PDF)

Table S1 **Fv/Fm values demonstrating the long-term stability of the** ***C. reinhardtii*** **strains under oxidative stress-related conditions.** Average values from two independent experiments are presented, ±SE, n = 4; n.s. – not survive; n.d. – not detected.
(PDF)

Table S2 **Free radical scavenging capacity of some** ***C. reinhardtii*** strains extracts determined by DPPH assay*. The value of *I_50_*, the concentration of algal extract (mg dry weight mL^−1^) that reduces 50% of the DPPH free radical, was estimated for each strain. TROLOX-Equivalent Antioxidant Capacity (TEAC) for each extract was calculated as ratio of algal *I_50_* and *I_50_* of the well-known antioxidant TROLOX (0.0032±0.0005 mg mL^−1^). Average values of 3 different cultures are presented, ±SE, n = 6. The values are significantly different from the reference strain at p≤0.05 (Mann-Whitney U Test).
(PDF)
