# Supplementary material for: A Powerful Molecular Engineering Tool Provided Efficient Chlamydomonas Mutants as Bio-Sensing Elements for Herbicides Detection
Source: PLoS One. 2013 Apr 17;8(4):e61851. doi: 10.1371/journal.pone.0061851 (PMC3629139; doi:10.1371/journal.pone.0061851)

Figure S1 Growth rate curves of the reference strain IL and D1 random mutants of *C. reinhardtii* selected for their PSII long-term stability under oxidative stress-related conditions. The time course of cell culture growth were followed for a period of 88 h under growth conditions by measuring: **A**) culture optical density (OD_{750}) and **B**) total chlorophyll content (μg mL^{-1}). Average values from four different cultures are presented, ±SE, n=4.

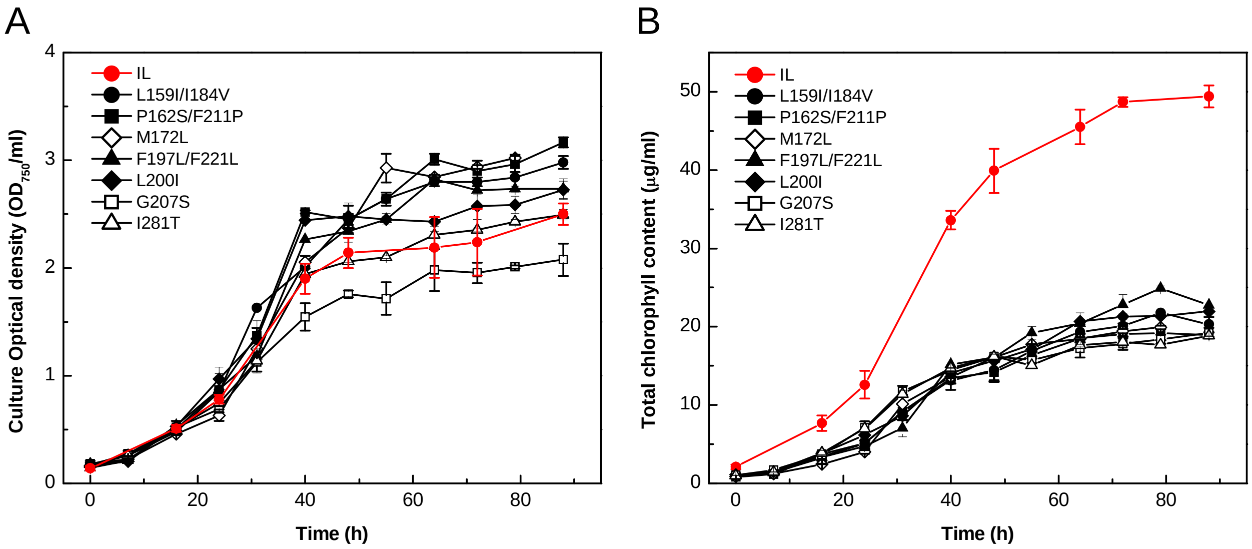

Supplement: Figure S1 — Growth rate curves of the reference strain IL and D1 random mutants of C. reinhardtii selected for their PSII long-term stability under oxidative stress-related conditions. The time courses of cell culture growth were followed for a period of 88 h under growth conditions by measuring: A) culture optical density (OD750) and B) total chlorophyll content (µg/ml). Average values from four different cultures are presented, ±SE, n = 4. (PDF) [file pone.0061851.s001.pdf]
