# Supplementary material for: A Powerful Molecular Engineering Tool Provided Efficient Chlamydomonas Mutants as Bio-Sensing Elements for Herbicides Detection
Source: PLoS One. 2013 Apr 17;8(4):e61851. doi: 10.1371/journal.pone.0061851 (PMC3629139; doi:10.1371/journal.pone.0061851)
Supplement: Table S1 — Fv/Fm values demonstrating the long-term stability of the C. reinhardtii strains under oxidative stress-related conditions. Average values from two independent experiments are presented, ±SE, n = 4; n.s. – not survive; n.d. – not detected. (PDF) [file pone.0061851.s002.pdf]

Table S1 Fv/Fm values demonstrating the long term stability of the *C. reinhardtii* strains under oxidative stress-related conditions. Average values from two independent experiments are presented, ±SE, n=4; n.s. – not survived; n.d. – not detected.

**Strains**	**Fv/Fm**	**Fv/Fm (% of the first day)**			
	on the 1st day of the treatment	30 days treatment	65 days treatment	80 days treatment	110 days treatment
IL	0.76 ± 0.01	78 ± 3	54 ± 5	43 ± 2	46 ± 4
L159I/I184V	0.70 ± 0.08	80 ±9	47 ± 4	n.d.	n.d.
L159M	0.74 ± 0.01	82 ± 2	55 ± 3	53 ± 1	57 ± 1
P162S/F211S	0.76 ± 0.02	88 ± 3	70 ± 4	n.d.	n.d.
M172L	0.75 ± 0.00	89 ± 1	66 ± 2	35 ± 12	n.s.
M172T	0.67 ± 0.00	3 ± 1	n.s.	n.s.	n.s.
S177P	0.72 ± 0.00	29 ± 5	4 ± 4	n.s.	n.s.
F197L/F285L	0.71 ± 0.07	80 ± 0	57 ± 3	n.d.	n.d.
L200I	0.77 ± 0.01	90 ± 2	73 ± 6	31 ± 10	n.s.
G207S	0.73 ± 0.02	86 ± 0	70 ± 2	n.d.	n.d.
I281T	0.76 ± 0.00	91 ± 0	79 ± 2	77 ± 1	75 ± 2
F274Y	0.76 ± 0.00	74 ± 1	25 ± 1	9 ± 2	n.s.
