# Supplementary material for: A Powerful Molecular Engineering Tool Provided Efficient Chlamydomonas Mutants as Bio-Sensing Elements for Herbicides Detection
Source: PLoS One. 2013 Apr 17;8(4):e61851. doi: 10.1371/journal.pone.0061851 (PMC3629139; doi:10.1371/journal.pone.0061851)
Supplement: Table S2 — Free radical scavenging capacity of some C. reinhardtii strains extracts determined by DPPH assay*. The value of I50, the concentration of algal extract (mg dry weight mL−1) that reduces 50% of the DPPH free radical, was estimated for each strain. TROLOX-Equivalent Antioxidant Capacity (TEAC) for each extract was calculated as ratio of algal I50 and I50 of the well-known antioxidant TROLOX (0.0032±0.0005 mg mL−1). Average values of 3 different cultures are presented, ±SE, n = 6. The values are significantly different from the reference strain at p≤0.05 (Mann-Whitney U Test). (PDF) [file pone.0061851.s003.pdf]

Table S2 Free radical scavenging capacity of some *C. reinhardtii* strains extracts determined by DPPH assay. The value of I_{50}, the concentration of algal extract (mg dry weight mL^{-1}) that reduces 50% of the DPPH free radical, was estimated for each strain. TROLOX-Equivalent Antioxidant Capacity (TEAC) for each extract was calculated as ratio of algal I_{50} and I_{50} of the well-known antioxidant TROLOX (0.0032 ± 0.0005 mg mL^{-1}). Average values of 3 different cultures are presented, ±SE, n=6. The values are significantly different from the reference strain at p≤0.05 (Mann-Whitney U Test).

Strains	I_{50} mg mL^{-1}	TEAC $I_{50ALGA}/I_{50TROLOX}$
IL	4.5 ± 0.1	1381
P162S/F211S	2.9 ± 0.2	883
M172L	3.9 ± 0.2	1205
L200I	3.8 ± 0.3	1171
G207S	3.0 ± 0.2	927
I281T	2.6 ± 0.4	803
F274Y	5.3 ± 0.3	1646

Experimental protocol

Cells in exponential growth phase were collected and lyophilized at -50 °C under vacuum overnight. After measuring the exact dry weight (DW) of each sample, the lyophilized material was re-hydrated with bi-distilled water overnight at 4°C. Subsequently, samples were centrifuged and pellet frozen in liquid nitrogen. The cells were broken in mortar adding liquid nitrogen and methanol (1 ml for each 10 mg of lyophilized material), in presence of $NaHCO_3$ to avoid pH drop due to cells lyses. Finally, the methanol extracts were centrifuged at 15000 g, at 4°C for 15 min, the supernatants were collected and stored at -20 °C until analysis.

The antioxidant activity, based on the extract capacity to scavenge the stable 2,2-diphenyl-1-picrylhydrazyl (DPPH) free radical, was determined as described by Huang et al., 2005. The reaction mixture consisted of 0.25 mL 0.25 mM DPPH in methanol and increasing volumes of cell extract (10 mg DW mL^{-1}) in final volume of 1 mL. The absorption of the reaction mixture was measured at 517 nm against methanol after 30 min incubation in room temperature and in

the dark. Upon reduction, the color of the solution fades. The antioxidant activity was calculated as percentage of the DPPH remaining, $\%DPPHrem=100\times(A_{517sample}/A_{517DPPH})$. Extracts I_{50} values were calculated by linear regression ($R^2>0.99$) of the graph plotting the %DPPHrem against the extract concentration (in mg mL^{-1}). The dose/response curve and I_{50} (mg mL^{-1}) value for the well-known antioxidant, TROLOX (water-soluble derivative of vitamin E, 6-hydroxy-2,5,7,8-tetramethylchroman-2-carboxylic acid, Sigma Aldrich) were also determined. The antioxidant activity of the algal extract and TROLOX compared.

Reference

Huang D, Ou B, Prior RL (2005) The chemistry behind antioxidant capacity assays. J Agric Food Chem 53: 1841–1856.
